# Propagation of the Israeli vaccine strain of *Anaplasma centrale* in tick cell lines

**DOI:** 10.1016/j.vetmic.2015.07.008

**Published:** 2015-09-30

**Authors:** Lesley Bell-Sakyi, Ana M. Palomar, Emma L. Bradford, Varda Shkap

**Affiliations:** a The Pirbright Institute, Ash Road, Pirbright, Woking, Surrey GU24 0NF, UK; b CIBIR, C/ Piqueras, 98, Logroño 26006, Spain; c Institute of Biological and Environmental Sciences, University of Aberdeen, Tillydrone Avenue, Aberdeen AB24 2TZ, UK; d Kimron Veterinary Institute, Bet Dagan, 50250, Israel

**Keywords:** Anaplasmosis, *Anaplasma centrale*, Vaccine, Tick cell line, *In vitro* culture

## Abstract

*Anaplasma centrale* has been used in cattle as a live blood vaccine against the more pathogenic *Anaplasma marginale* for over 100 years. While *A. marginale* can be propagated *in vitro* in tick cell lines, facilitating studies on antigen production, immunisation and vector-pathogen interaction, to date there has been no *in vitro* culture system for *A. centrale*. In the present study, 25 cell lines derived from 13 ixodid tick species were inoculated with the Israeli vaccine strain of *A. centrale* and monitored for at least 12 weeks by microscopic examination of Giemsa-stained cytocentrifuge smears. Infection of 19 tick cell lines was subsequently attempted by transfer of cell-free supernate from vaccine-inoculated tick cells. In two separate experiments, rickettsial inclusions were detected in cultures of the *Rhipicephalus appendiculatus* cell line RAE25 28–32 days following inoculation with the vaccine. Presence of *A. centrale* in the RAE25 cells was confirmed by PCR assays targeting the 16S rRNA, *groEL* and *msp4* genes; sequenced PCR products were 100% identical to published sequences of the respective genes in the Israeli vaccine strain of *A. centrale*. *A. centrale* was taken through three subcultures in RAE25 cells over a 30 week period. In a single experiment, the *Dermacentor variabilis* cell line DVE1 was also detectably infected with *A. centrale* 11 weeks after inoculation with the vaccine. Availability of an *in vitro* culture system for *A. centrale* in tick cells opens up the possibility of generating a safer and more ethical vaccine for bovine anaplasmosis.

## Highlights

• First *in vitro* culture system for *Anaplasma centrale.*
• *A. centrale* infected and grew in two out of 32 tick cell lines tested.
• Potential for safer and more ethical bovine anaplasmosis vaccine.

## 1 Introduction

*Anaplasma centrale*, first isolated from a heifer in South Africa in 1909 (Theiler, 1911; cited by Herndon et al., 2013), has been used as a live blood vaccine to protect against bovine anaplasmosis caused by *Anaplasma marginale* for over 100 years. Currently the *A. centrale* vaccine is used to protect cattle in several African, South American and Middle Eastern countries including Israel. Production of the vaccine involves infecting splenectomised cattle with *A. centrale* stabilate and harvesting large volumes of blood from them when the rickettsaemia reaches a suitable level (OIE, 2014). Live blood vaccines have a number of disadvantages including risk of co-transmission of other ruminant pathogens, risk of haemolytic disease in calves born to vaccinated dams and requirement for a stringent cold chain. While an *in vitro* culture system for *A. marginale* in cell lines derived from the tick *Ixodes scapularis* has been available for nearly two decades (Munderloh et al., 1996), and has resulted in exponential progress in knowledge and understanding of this pathogen, to date it has not been possible to propagate *A. centrale in vitro*. Ability to cultivate *A. centrale in vitro* would open up the possibility of producing vaccine antigen without the need to splenectomise, infect and exsanguinate cattle.

The present study was carried out with the aim of establishing *in vitro* culture of the Israeli vaccine strain of *A. centrale* in one or more tick cell lines, taking advantage of the availability in the Tick Cell Biobank (http://www.pirbright.ac.uk/research/Tickcell/Default.aspx) of multiple cell lines derived from five ixodid tick genera.

## 2 Materials and methods

### 2.1 Tick cell lines

A panel of 32 tick cell lines derived from 14 ixodid tick species (Table 1) were tested for ability to support infection and replication of *A. centrale*. The cell lines were grown at either 28 °C or 32 °C in sealed flat-sided culture tubes (Nunc) containing 2.2 ml of complete culture medium (L-15, H-Lac, L-15B, L-15B300, L-15/MEM, L-15/H-Lac, L-15/L-15B or L-15/H-lac/L-15B as described previously (Munderloh and Kurtti, 1989; Munderloh et al., 1999; Bell-Sakyi, 2004). Prior to infection with *A. centrale* the supernatent medium was removed from each tube, the cell monolayer was washed once with 1 ml of L-15B medium supplemented with 10% FCS, 10% TPB, 0.1% bovine lipoprotein (MP Biomedicals), 2 mM L-glutamine, 15 mM HEPES and 0.1% NaHCO_3_ (ACGM) to remove traces of antibiotics and 2 ml of ACGM was added to the tube. For cultures receiving blood vaccine, ACGM was further supplemented with 5 μg/ml Amphotericin B (ACGMA).

### 2.2 Inoculation of tick cell lines with *Anaplasma centrale-*infected bovine erythrocytes

The Israeli *A. centrale* blood vaccine comprising bovine erythrocytes with *A. centrale* rickettsaemia of 20%, cryopreserved with 5% DMSO as 1.8 ml aliquots containing 1 × 10^8^ infected erythrocytes, was prepared at the Kimron Veterinary Institute and stored in the vapor phase of a liquid nitrogen refrigerator prior to and following transfer on dry ice to the Pirbright Institute. For inoculation onto tick cell lines, a vial of vaccine was thawed rapidly by immersion in a 37 °C water bath and the contents were immediately diluted in 9 ml of ACGMA at room temperature. Aliquots of 0.6–0.7 ml were immediately added to tubes of tick cells in ACGMA, the contents of each tube was mixed by gentle rocking 2–3 times, and the cultures were incubated at 28 °C or 32 °C.

### 2.3 Maintenance and light microscopical analysis of tick cell lines inoculated with *A. centrale*

The medium of inoculated cultures was changed after 48 h by removal and replacement of 1.0–1.5 ml ACGMA. Thereafter, medium was changed weekly and, after the second week, ACGMA was replaced with ACGM. Cultures were examined weekly for at least 12 weeks by inverted microscope prior to the medium change for evidence of microbial contamination, general cell health and signs of *Anaplasma* infection. Giemsa-stained cytocentrifuge smears were prepared at 2–3 week intervals from approx. 50 μl of resuspended cells and examined at 500× and 1000× (oil immersion) for presence of *A. centrale* bacteria. Photomicrographs were taken using a CCD digital camera attached to a Zeiss Axioskop microscope and Zeiss Axiovision software.

### 2.4 Subculture of *A. centrale* within and between tick cell lines

Subcultures were carried out onto a fresh cell culture of the same tick cell line by transfer of 0.3–0.5 ml of supernatent medium without centrifugation. For subculture into different tick cell lines, supernatent medium from tick cell cultures previously inoculated with material containing *A. centrale* was clarified by centrifugation at 1500 × *g* for 5 min to remove intact cells, and 0.3–0.5 ml of clarified supernate was added to fresh cultures of the recipient cell line. All subcultures were incubated at 32 °C regardless of the normal incubation temperature of the recipient cell line.

## 3 Molecular analysis

DNA was extracted from residual *A. centrale* blood vaccine and from tick cell cultures using a DNeasy blood and tissue kit (Qiagen) following the manufacturer’s instructions for Gram-negative bacteria. DNA extracts were used as templates for PCRs targeting fragments of the 16S rRNA and *groEL* (HSP60) genes for *Ehrlichia* and *Anaplasma* detection (Schouls et al., 1999; Lew et al., 2003). Furthermore, a multiplex PCR assay for *A. centrale* and *A. marginale msp4* gene fragments (Shkap et al., 2008) was also performed. Lastly, a PCR specific for amplification of the *A. marginale msp1-α* gene (Shkap et al., 2002) was carried out. A negative control containing water instead of template DNA was included in all PCRs. PCR primer pairs, sizes of the amplicons (bp) and annealing temperatures used in the assays are shown in Table 2. All the PCRs were performed as described by the respective authors.

Positive PCR products were purified using a High Pure PCR Product Purification kit (Roche Life Science) following the manufacturer’s instructions. Purified amplification products were sequenced in the forward and reverse directions, and homology searches were performed in the NCBI database using the BLAST search programme (http://blast.ncbi.nlm.nih.gov/Blast.cgi). Sequences were aligned using the European Bioinformatics Institute multisequence software ClustalW2 (http://www.ebi.ac.uk/Tools/msa/clustalw2) for multiple sequence alignment.

## 4 Results

### 4.1 Isolation of *centrale* from blood vaccine inoculum

**Fig. 1:**
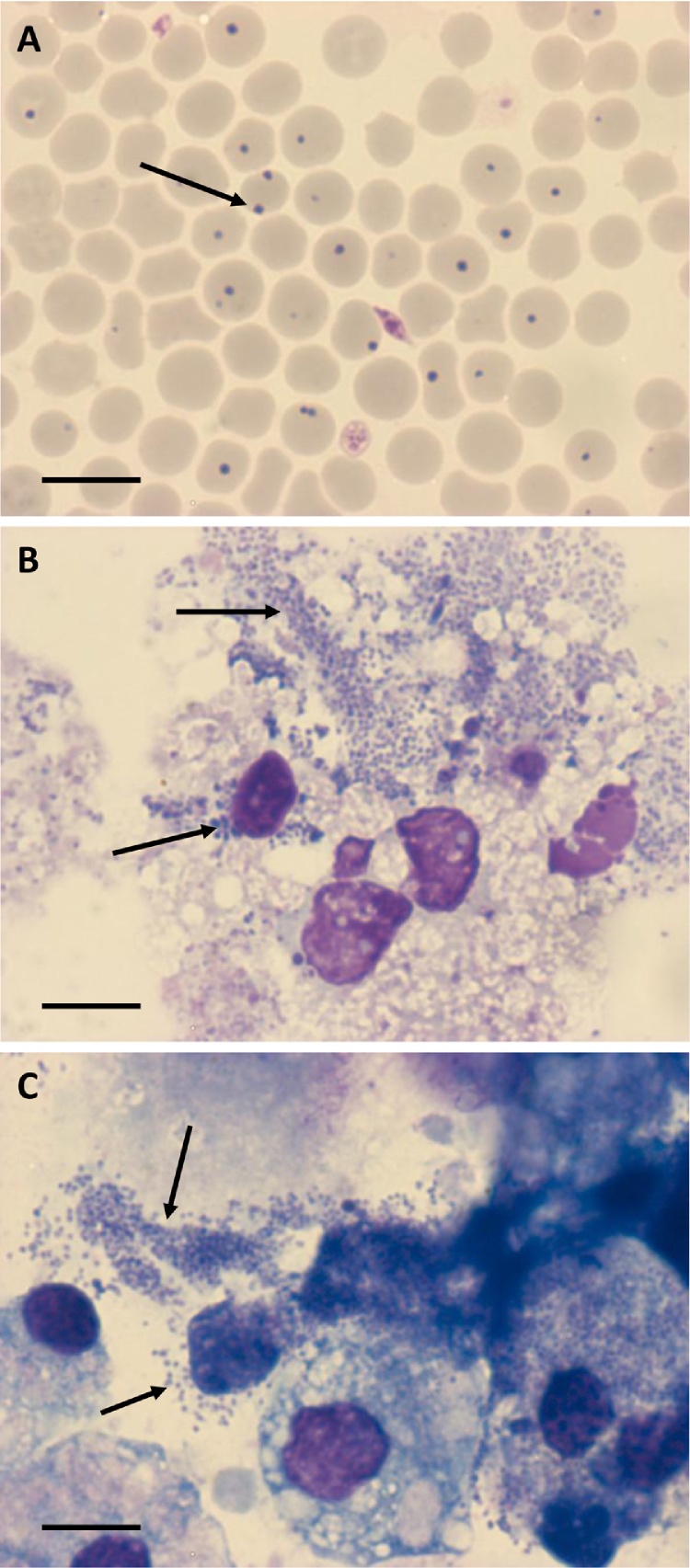
*Anaplasma centrale* in Giemsa-stained cytocentrifuge smears of (A) thawed, undiluted blood vaccine, (B) RAE25 cells 122 days post inoculation and (C) DVE1 cells 88 days post inoculation. Arrows indicate bacteria. Scale bar = 10 μm.

Twenty-five tick cell lines (Table 1) were inoculated with thawed, diluted *A. centrale* blood vaccine derived from one of two different cryovials in separate experiments. Cytocentrifuge smears prepared from undiluted inoculum showed numerous intrerythrocytic rickettsial bodies (Fig. 1A). Of the tick cell lines, all survived the inoculation except BME/CTVM6 in which all cells died within 24 h. In all cases, the inoculum formed a loose plasma clot incorporating some of the tick cells from the monolayer; this clot did not appear to have any deleterious effect on the cultures, and was gradually broken up during resuspension for preparation of cytocentrifuge smears.

Only two of the 25 tick cell lines showed evidence of *A. centrale* infection during the subsequent 12-week observation period: *Rhipicephalus appendiculatus* embryo-derived RAE25 and *Dermacentor variabilis* embryo-derived DVE1, both incubated at 32 °C. Small numbers of cell-free or cell-associated atypical *Anaplasma*-like bacteria were seen in Giemsa-stained cytocentrifuge smears of RAE25 cells inoculated with diluted vaccine from both cryovials after 28–32 days incubation. These gradually became more numerous and increasingly typical in appearance over the subsequent weeks (Fig. 1B). By 12 weeks post inoculation, the cultures were heavily infected and dead and dying cells and abundant extracellular debris were visible by inverted microscope examination of cultures, and by 18 weeks many, though not all, of the RAE25 cells were dead. Typical *A. centrale* bacterial inclusions were first seen in a few DVE1 cells inoculated with diluted vaccine from one of the two cryovials after 11 weeks in culture. Infected cells (Fig. 1C) gradually became more numerous, reaching an infection rate of 5% at 6 months post inoculation.

### 4.2 Confirmation of *A. centrale* identity by PCR

**Fig. 2:**
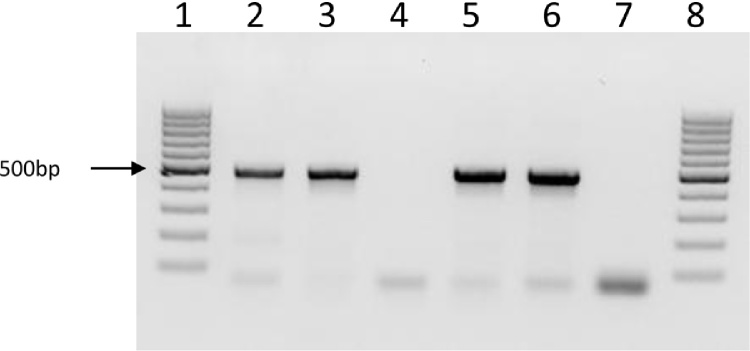
PCR amplification using primers targeting a 468 bp fragment of the 16S rRNA gene with a sequence conserved between *Anaplasma* and *Ehrlichia* spp. DNA extracted on day 42 post inoculation. Lanes 1 and 8: molecular weight markers; lane 2: positive control (*Ehrlichia ruminantium* DNA); lane 3: inoculum (*Anaplasma centrale* Israeli blood vaccine); lane 4: uninfected RAE25 cells; lane 5: RAE25 cells inoculated with *A. centrale*; lane 6: IDE8 cells inoculated with *A. centrale*; lane 7: negative control (no DNA).

**Fig. 3:**
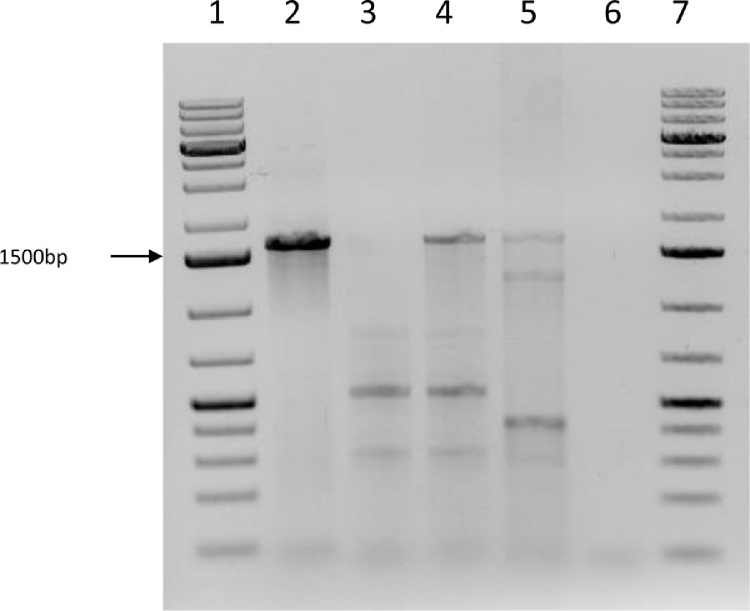
PCR amplification using primers targeting a 1650 bp fragment of the *groEL* gene of *Anaplasma centrale*. DNA extracted on day 42 post inoculation. Lanes 1 and 7: molecular weight markers; lane 2: inoculum (*A. centrale* Israeli blood vaccine); lane 3: uninfected RAE25 cells; lane 4: RAE25 cells inoculated with *A. centrale*; lane 5: IDE8 cells inoculated with *A. centrale*; lane 6: negative control (no DNA).

**Fig. 4:**
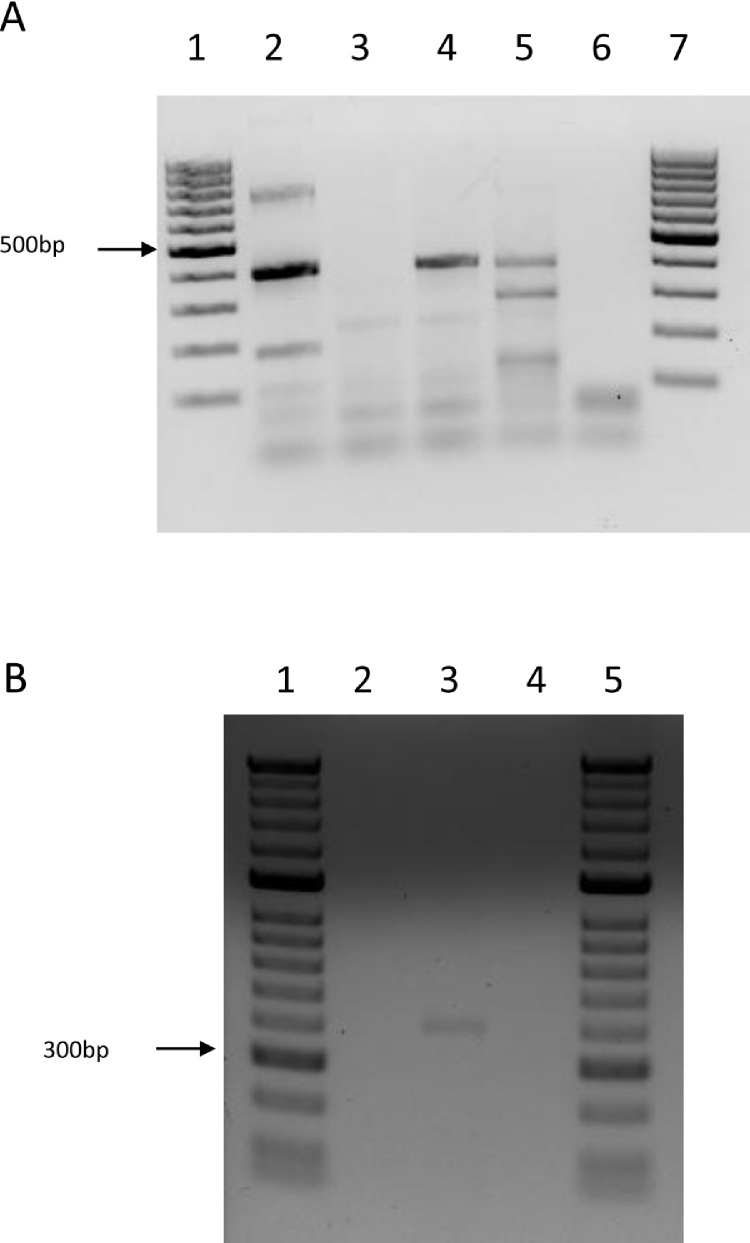
Multiplex PCR amplification using primers targeting fragments of the *msp4* genes of *Anaplasma marginale* (761 bp) and *Anaplasma centrale* (396 bp). A. DNA extracted on day 42 post inoculation. Lanes 1 and 7: molecular weight markers; lane 2: inoculum (*A. centrale* Israeli blood vaccine); lane 3: uninfected RAE25 cells; lane 4: RAE25 cells inoculated with *A. centrale* blood vaccine; lane 5: IDE8 cells inoculated with *A. centrale* blood vaccine; lane 6: negative control (no DNA). B. DNA extracted 6 months post inoculation. Lanes 1 and 5: molecular weight markers; lane 2: uninfected DVE1 cells; lane 3: DVE1 cells inoculated with *A. centrale* blood vaccine; lane 4: negative control (no DNA).

At 6 weeks post inoculation, DNA was extracted from *Anaplasma*-positive and control uninoculated RAE25 cells, from *Anaplasma*-inoculated IDE8 cells (an *I. scapularis* embryo-derived cell line that supports growth of other *Anaplasma* spp. but in which there was no evidence of replicating bacteria in Giemsa-stained smears) and from residual *A. centrale* blood vaccine stored at −20 °C in the original cryovials since the day of inoculum preparation. PCR amplification of a fragment of the 16S rRNA gene produced bands of the expected size (468 bp) in the blood vaccine and in the RAE25 and IDE8 cells previously inoculated with diluted *A. centrale* blood vaccine (Fig. 2). PCR amplification of a fragment of a second *Anaplasma* gene, *groEL*, from the same samples produced a very strong band of the expected size (1650 bp) in the vaccine, a fainter band in the *A. centrale*-inoculated RAE25 cells, and a very faint band in the *A. centrale*-inoculated IDE8 cells (Fig. 3). A multiplex PCR targeting the *msp4* genes of both *A. centrale* and *A. marginale* produced bands of the expected size for *A. centrale* in vaccine and *A. centrale*-inoculated RAE25 and IDE8 cells in decreasing order of strength, and a very faint band of the expected size for *A. marginale* in the blood vaccine (Fig. 4A). The PCR targeting the *msp1-a* gene of *A. marginale* failed to amplify any PCR product from the vaccine or the *A. centrale*-inoculated RAE25 and IDE8 cells (data not shown), indicating that the *A. centrale* blood vaccine was not contaminated with *A. marginale*. The multiplex PCR was also used at 6 months post inoculation to confirm that the bacteria seen in DVE1 cells inoculated with blood vaccine were *A. centrale* (Figure 4B). There was no amplification of specific products of the expected size from the uninfected RAE25 or DVE1 cells in any of the PCR assays.

All the sequences obtained from the 16S rRNA (426 bp), *groEL* (1545 bp) and *A. centrale msp4* (357 bp) PCR products were identical to each other for each gene, and showed 100% identity with the sequences corresponding to the *A. centrale* Israeli vaccine strain deposited in GenBank (Table 3). The 16S rRNA sequences were also 100% identical to those of the Australian *A. centrale* vaccine strain and *A. centrale* isolated from *Rhipicephalus simus* ticks in South Africa (Potgieter and Van Rensberg, 1987), while the *groEL* sequences were 100% similar to the Australian vaccine stain and 99.2% similar to the *R. simus*-derived strain (Table 3). Unexpectedly, the sequence of the amplicon of the expected size for *A. marginale msp4* (703 bp) obtained from the blood vaccine in the multiplex PCR was also homologous to the *A. centrale msp4* sequence (Table 3), indicating that the *A. centrale* vaccine was not contaminated with *A. marginale*. For all three genes examined, levels of similarity with published sequences of *A. marginale* strains from Israel, USA and Australia were lower than with the *A. centrale* strains: 99.1% for 16S rRNA, 97.1–97.4% for *groEL* and 80.1–83.1% for *msp4* (Table 3).

### 4.3 *A. centrale* subculture and transfer between different tick cell lines

*A. centrale* from RAE25 cells was successfully subcultured into fresh RAE25 cells 64 days after culture initiation and, at the time of writing, has been maintained in RAE25 cells through three passages over a 210 day-period. Cell-free supernate from infected RAE25 cells was inoculated onto 18 heterologous tick cell lines (Table 1) including 10 cell lines that failed to become infected following inoculation with *A. centrale* blood vaccine. Of these, only DVE1 cells became detectably infected with *A. centrale* during the 12-week observation period, as determined by microscopic examination.

## 5 Discussion

For the first time, *A. centrale* has been successfully propagated *in vitro* in tick cell lines derived from the ixodid species *R. appendiculatus* and *D. variabilis*. Amplification and sequencing of three *Anaplasma* genes confirmed that the bacteria growing in the tick cell lines was indeed *A. centrale* and not *A. marginale*. Interestingly, isolation from intraerythrocytic stages of *A. centrale* was not achieved in any of the cell lines derived from *I. scapularis* ticks that have been successfully used to isolate and cultivate a wide range of intracellular arthropod-borne bacteria of the genera *Anaplasma*, *Ehrlichia* and *Rickettsia* (reviewed by Bell-Sakyi et al., 2007), *Cardinium* (Kurtti et al., 1996) and *Neoehrlichia* (Munderloh et al., 2007). While vaccine-inoculated IDE8 cells were PCR-positive 6 weeks post inoculation, presumably as a result of residual bacterial DNA or non-viable bacteria as reported for tick cell lines inoculated with mammalian stages of *Ehrlichia ruminantium* (Bell-Sakyi, 2004), no microscopic evidence of bacterial replication was seen at any time in any of the *I. scapularis* cell lines inoculated with either *A. centrale* blood vaccine or with *A. centrale* transferred from infected RAE25 cells.

The *R. appendiculatus* cell line RAE25 supports growth of several tick-borne bacteria including *E. ruminantium* (Bell-Sakyi, 2004), *Ehrlichia canis*, *Ehrlichia mineirensis* and *Anaplasma phagocytophilum* (author’s unpublished results) and a spotted fever group *Rickettsia* (Munderloh et al., 1998), but has not been reported previously to be a suitable cell line for isolation of Anaplasmataceae bacteria from mammalian cells. The *D. variabilis* cell line DVE1 supports growth of *Rickettsia peacockii* (Kurtti et al., 2005) and *E. canis* (author’s unpublished results); in both cases the bacteria were transferred from already-infected cell lines derived from different tick species, making the present study the first report of successful isolation of an intracellular bacterium directly from mammalian cells into DVE1. In contrast to *E. ruminantium* which, once established in IDE8 cells was readily transferred into heterologous tick cell lines refractory to infection with mammalian stages of the bacterium (Bell-Sakyi, 2004), in the present study it was only possible to transfer *A. centrale* infection between tick cell lines that were susceptible to infection with the intraerythrocytic stages.

Natural tick transmission of *A. centrale* has only been reported once; a previously tick-free splenectomised ox infested with 80 adult *R. simus* collected in the field in South Africa contracted *A. centrale* infection (Potgieter and Van Rensberg, 1987). Descendants of the same ticks (120–130 adults infected as nymphs) transstadially transmitted *A. centrale* between splenectomised cattle (*n* = 2), although it is unclear whether this was the field isolate of *A. centrale* or the vaccine strain. A single splenectomised ox inoculated with ground-up unfed adult *R. simus*, previously fed as nymphs on a calf that had contracted the *A. centrale* vaccine strain *in utero*, developed patent *A. centrale* infection. The authors failed to transmit *A. centrale* between splenectomised cattle by interrupted feeding of the one-host ticks *Rhipicephalus* (*Boophilus*) *decoloratus* and *Rhipicephalus* (*Boophilus*) *microplus* (Potgieter and Van Rensberg, 1987).

More recently, Ueti et al. (2007) demonstrated that when adult male *Dermacentor andersoni* ticks were acquisition-fed for 7 days on *A. centrale*-infected cattle, incubated off-host for a further 7 days and then transmission-fed, 54% of midguts and 71% of salivary glands from 150 ticks were PCR-positive for *A. centrale*. However, presence of *A. centrale* bacteria in these organs was not demonstrated microscopically, and 100 of these ticks failed to transmit *A. centrale* to either of two susceptible calves. In a subsequent study, Herndon et al. (2013) reported transmission of *A. centrale* between calves by batches of 500 adult male *D. andersoni* acquisition-fed as above, while batches of 100 ticks failed to transmit the pathogen, indicating that male *D. andersoni* ticks were not efficient vectors of *A. centrale* despite apparently supporting bacterial infection of midgut and salivary glands.

Shkap et al. (2009) failed to experimentally transmit the Israeli vaccine strain of *A. centrale* by interrupted feeding (as above) of any of adult male or female *Hyalomma excavatum*, *Rhipicephalus* (*Boophilus*) *annulatus* or *Rhipicephalus sanguineus* ticks, although *A. centrale* DNA was detected in salivary glands of 1/10 *R. sanguineus* ticks transmission fed for 6 days.

In the present study, cell lines derived from five *Rhipicephalus* species (including two *R. sanguineus* cell lines, RSE8 and RML-RSE) and four *Dermacentor* species (including two *D. andersoni* cell lines, DAE15 and DAE100T) were tested for ability to support *A. centrale* infection and growth. Of these, there was no evidence of infection in the cell lines derived from tick species previously reported to be experimentally infectable with *A. centrale* – RSE8, RML-RSE, DAE15 and DAE100T – while one out of four *R. appendiculatus* and one *D. variabilis* cell lines did become infected. As far as we know, neither *R. appendiculatus* nor *D. variabilis* have been tested for ability to transmit *A. centrale*; the results of the present study suggest that the vector capacity of these two tick species should be investigated. As there is no cell line derived from *R. simus*, it was not possible to compare the *in vitro* susceptibility of cells from this tick species with those of *R. appendiculatus*.

Availability of continuous *in vitro* culture systems for other *Anaplasma* species resulted in a rapid expansion of knowledge on many different aspects of, in particular, *A. marginale* and *A. phagocytophilum*, reviewed by Blouin et al. (2002), Bell-Sakyi et al. (2007) and more recently by Passos (2012). The ability to grow *A. centrale* in tick cells will facilitate a similar expansion of knowledge of this enigmatic bacterium and, importantly, opens up the possibility of producing *A. centrale* vaccine in an *in vitro* system that will be much safer and more controllable than the current *in vivo* system (OIE, 2014). Following this vital but preliminary step of establishing the *in vitro* culture system, further research will be needed to determine the safety, immunogenicity and efficacy of tick cell-derived *A. centrale* as a putative vaccine antigen.

## Figures and Tables

**Table 1:** Tick cell lines tested for ability to support growth of *Anaplasma centrale*. The original references for the tick cell lines are cited by Alberdi et al. (2012) except where indicated. *A. centrale* was inoculated (X) as either diluted vaccine or as clarified supernate from already-infected tick cells.

Tick species	Cell line	Culture medium/incubation temperature	Inoculum	
			From vaccine	From tick cells
*Amblomma americanum*	AAE2	L-15B300/32 °C		X
	AAE12	L-15B300/32 °C		X

*Amblyomma variegatum*	AVL/CTVM13	L-15/L-15B/32 °C		X
	AVL/CTVM17	L-15/H-Lac/L-15B/32 °C	X	

*Dermacentor albipictus*	DALBE3	L-15B300/32 °C	X	

*Dermacentor andersoni*	DAE15	L-15B300/32 °C	X	X
	DAE100T	L-15B300/32 °C	X	X

*Dermacentor nitens*	ANE58	L-15B300/32 °C	X	
*Dermacentor variabilis*	DVE1	L-15B300/32 °C	X	X

*Hyalomma anatolicum*	HAE/CTVM8	L-15/H-Lac/32 °C		X
	HAE/CTVM9	L-15/MEM/32 °C	X	

*Ixodes ricinus*	IRE/CTVM19	L-15/28 °C	X	

*Ixodes scapularis*	IDE2	L-15B300/32 °C	X	X
	IDE8	L-15B/32 °C	X	X
	ISE6	L-15B300/32 °C	X	X
	ISE18	L-15B300/32 °C	X	

*Rhipicephalus appendiculatus*	RAE/CTVM1	L-15/28 °C	X	X
	RAN/CTVM3	H-Lac/28 °C		X
	RAE25^a^	L-15B/32 °C	X	X
	RA243	L-15/32 °C	X	X

*Rhipicephalus evertsi*	REE/CTVM29	L-15/28 °C		X
	REE/CTVM31	L-15/MEM/28 °C	X	
	REN/CTVM32^b^	L-15/H-Lac/28 °C	X	

*Rhipicephalus sanguineus*	RSE8	L-15/L-15B/32 °C		X
	RML-RSE^c^	L-15/MEM/28 °C	X	

*Rhipicephalus* (*Boophilus*) *decoloratus*	BDE/CTVM16	L-15/28 °C	X	X

*Rhipicephalus* (*Boophilus*) *microplus*	BME/CTVM2	L-15/28 °C	X	X
	BME/CTVM5	L-15/MEM/28 °C	X	
	BME/CTVM6	L-15/28 °C	X	
	BME/CTVM23	L-15/32 °C	X	X
	BME/CTVM30	L-15/MEM/28 °C	X	
	BmVIII-SCC	L-15/MEM/32 °C	X	

a Kurtti and Munderloh (1982).

b Bell-Sakyi (unpublished); derived from developing adult *R. evertsi* ticks kindly supplied in 2010 by Dr. Ard Nijhof, then of Utrecht Centre for Tick-borne Diseases, Utrecht University, The Netherlands.

c Previously deposited in the Tick Cell Biobank as *D. variabilis* embryo-derived cell line RML-15 (Yunker et al., 1981). However sequencing of a fragment of the 16S rRNA gene (Black and Piesman, 1994) revealed that the cell line was actually derived from *R. sanguineus* (data not shown). Three embryo-derived *R. sanguineus* cell lines were established in the same laboratory as RML-15: RML-21, 22 and 23 (Yunker et al., 1984, 1987). As it is now impossible to determine which of the three cell lines was used in the present study, it is here designated RML-RSE.

**Table 2:** PCR primer pairs and conditions used in this study. W = T or A; Y = T or G; R = G or A; M = A or C; N = G or A or T or C.

Target organism	Target gene	Primer sequence 5′ → 3′	Amplicon size (bp)	Annealing temp. (°C)	Reference
*Ehrlichia*/*Anaplasma*	16S rRNA	16S8FE: GGAATTCAGAGTTGGATCMTGGYTCAG B-GA1B: CGGGATCCCGAGTTTGCCGGGACTTCTTCT	468	67–57 (touchdown)	Schouls et al., 1999
	*groEL*	HSPC: AAATGGCGAATGTTGTWGTYAC HSPB: TTARAARCCRCCCATRCCRCCCATGCC	1650	60	Lew et al., 2003
	
*A. centrale*	msp4*	F: CATGGGGCATGAATCTGTG R: AATTGGTTGCAGTGAGCGC	395	53	Shkap et al., 2008

*A. marginale*	msp4*	F: CATCTCCCATGAGTCACGAAGTGGC R: GCTGAACAGGAATCTTGCTCC	761	53	Shkap et al., 2008
	*msp1α*	F: GCATTACAACGCAACGCTT R: ACCTTGGAGCGCATCTCTT	515-687	56	Shkap et al., 2002

*Both genes are targeted in the same multiplex PCR.

**Table 3:** Similarity of the partial genes amplified in the present study with *Anaplasma centrale* and *Anaplasma marginale* sequences deposited in GenBank.

*Anaplasma* spp. (GenBank accession no.)	16S rRNA % identity (bp)	*groEL* % identity (bp)	*msp4* % identity (bp)	
			Small amplicon	Large amplicon
*A. centrale* str. Israel (CP001759)	100 (426/426)	100(1545/1545)	100(357/357)	100(703/703)
*Anaplasma centrale* strain vaccine from Australia (AF414868, AF414867)	100 (426/426)	100(1545/1545)	–	–
*A. centrale* from South Africa from *Rhipicephalus simus* (AF414869, AF414866)	100 (426/426)	99.2(1532/1545)	–	–
*A. marginale* from Israel non-tailed (AF414875, AF414861, AY786993)	99.1(422/426)	97.3(1503/1545)	82.1(293/357)	81.8(570/703)
*A. marginale* from Israel tailed (AF414876, AF414862, AY786994)	99.1(422/426)	97.3(1503/1545)	82.1(293/357)	80.1(568/703)
*A.marginale* strain St. Maries from USA (CP000030)	99.1(422/426)	97.1(1500/1545)	82.1(293/357)	81.2(571/703)
*A.marginale* strain Florida from USA (CP001079)	99.1(422/426)	97.2(1501/1545)	82.4(294/357)	81.4(572/703)
*A. marginale* strain Gypsy Plains from Australia (CP006846)	99.1(422/426)	97.4(1505/1545)	82.1(293/357)	83.1(584/703)
*A.marginale* strain Dawn from Australia (CP006847)	99.1(422/426)	97.4(1505/1545)	82.1(293/357)	81.5(573/703)
